# The geography of technological innovation dynamics

**DOI:** 10.1038/s41598-023-48342-8

**Published:** 2023-11-29

**Authors:** Matteo Straccamore, Vittorio Loreto, Pietro Gravino

**Affiliations:** 1 Physics Department, Sapienza Univ. of Rome, Piazzale Aldo Moro 2, 00185 Rome, Italy; 2 Centro Ricerche Enrico Fermi, Via Panisperna 89/A, 00184 Rome, Italy; 3 Sony CSL - Rome, Joint Initiative CREF-SONY, Centro Ricerche Enrico Fermi, Via Panisperna 89/A, 00184 Rome, Italy; 4 Sony CSL - Paris, 6, Rue Amyot, 75005 Paris, France; 5 Complexity Science Hub, Josefstädter Strasse 39, 1080 Vienna, Austria

**Keywords:** Complex networks, Environmental social sciences

## Abstract

Cities and metropolitan areas are major drivers of creativity and innovation in all possible sectors: scientific, technological, social, artistic, etc. The critical concentration and proximity of diverse mindsets and opportunities, supported by efficient infrastructures, enable new technologies and ideas to emerge, thrive, and trigger further innovation. Though this pattern seems well established, geography’s role in the emergence and diffusion of new technologies still needs to be clarified. An additional important question concerns the identification of the technological innovation pathways of metropolitan areas. Here, we explore the factors that influence the spread of technology among metropolitan areas worldwide and how geography and political borders impact this process. Our evidence suggests that political geography has been highly important for the diffusion of technological innovation till around two decades ago, slowly declining afterwards in favour of a more global patenting ecosystem. Further, the visualisation of the evolution of countries and metropolitan areas in a 2d space of competitiveness and diversification reveals the existence of two main technological innovation pathways, discriminating between different strategies towards progress. Our work provides insights for policymakers seeking to promote economic growth and technological advancement through tailored investments in prioritarian technological innovation areas.

## Introduction

In our increasingly interconnected world, diffusion processes play a crucial role in determining the evolution of our societies. For this reason, a well-established and growing literature is focusing on studying the different instances of the phenomenon, from information diffusion in social networks^1,2^ to the spreading of diseases^3–5^. Particular attention converged on the diffusion of innovations^6,7^ and technologies^8–10^. The adoption of patent data to monitor technological innovation is well established^11–13^. For the past few decades, patent data have become a workhorse for the literature on technical change, due mainly to the growing availability of data about patent documents^14^. This ever-increasing data availability (e.g., PATSTAT, REGPAT and Google Patents^15^) has facilitated and prompted researchers worldwide to investigate various questions regarding the patenting activity. For example, on the nature of inventions, their network structure, and their role in explaining technological change^14,16,17^. One of the characteristics of patent documents is the presence of codes associated with the claims in patent applications. These codes mark the boundaries of the commercial exclusion rights demanded by inventors. Claims are classified based on the technological areas they impact, according to existing classifications (e.g., the IPC classification^18^), to allow the evaluation by patent offices. Mapping claims to classification codes allows localizing patents and patent applications within the technology “semantic” space^19^.

In addition to the semantic space defined through technological codes, patents and technological innovation live in a physical space. It is known, for instance, the role that cities and metropolitan areas play in fostering creativity and innovation. Thanks to a critical concentration and proximity of diverse mindsets and opportunities, urban infrastructures enable new technologies and ideas to emerge, thrive, and trigger further innovation. Still, more is needed to know about the interplay between geography’s role and the innovation processes’ semantics. Technological innovation diffusion processes take place, in fact, in a geographical layer that still needs to be studied, both from the physical and political points of view.

However, it is essential to highlight that the only use of patents as a proxy for innovation^20^ could be restrictive. Inventions do not represent all forms of knowledge production in the economy, nor do patents cover all generated knowledge^21^, and assessing their value is not always straightforward^22^. In addition to technological innovation and patents, several studies identify other aspects of innovation. Rutten^23^ identifies four cross-case mechanisms that explain regional innovation: diversity, cosmopolitan environment, technology transfer, and creativity. Also, Filippopoulos et al.^24^ examine regional innovation in Europe, and they identify various mechanisms that contribute to regional innovation: business and public sector R &D, proximity to external R &D, collaboration networks, tolerance, inclusion, and human capital. Moreover, they show how technological innovation only appears in more developed regions that present business R &D, internal R &D competence, and tolerance/inclusion, with potential support from public R &D. Still, both studies highlight technological innovation as one of the most important aspects of innovation. In this study, we decided to focus on developed metropolitan areas for which technological innovation is a key driver of innovation.

Cities and metropolitan areas (MAs) appear thus as the right level to investigate the role of geography in innovation processes. To date, approximately 55% of the global population lives in urban areas, which represent the core of innovation^25,26^, economy^27^, science^28^, and much more. According to a report by the World Bank^29^, MAs generate about 80% of global GDP. They attract businesses and industries, creating jobs and driving innovation^30^; also, from an environmental perspective, MAs can be more sustainable than rural areas due to their greater efficiency in resource use and transportation^31^. For all these reasons, we focus on metropolitan areas as the smallest geographical entities, after countries and regions, essential for economic growth and development. However, authors such as Shearmur^32^ criticise that innovation is intimately tied to cities and clusters of economic activity. In his words, “The geography of innovation- as an area of study-does not seriously examine innovation by isolated firms or in remote areas, which it considers atypical.” He argues that the evidence upon which this assumption is based is biased toward identifying innovation in clusters and urban areas, and that innovation theory contributes to this bias. Though we agree that non-urban areas could be relevant in boosting innovation, here we limit our analysis to developed metropolitan areas with intense patenting activity.

Many recent studies have relied on network-based techniques to unfold the complex interplay among patents, technological codes, and geographical reference areas. We decided to use the framework of bipartite networks^33^, which are suitable whenever systems involve interactions between pairs of entities. For example, in ecology, interactions between two types of species can be described using bipartite networks, such as plant-pollinator networks^34^ or seed-disperser networks^35^. Bipartite networks are also used in social^36^, economic^37–39^, and biological^40^ systems.

With the tools described above and a specific focus on metropolitan areas, this paper investigates the factors that influence the spread of technology among metropolitan areas worldwide and how geography and political borders impact this process. We reveal that the current technological innovation pathways can be effectively predicted if one considers a non-trivial interplay between, on the one hand, the similarity between the technological content of cities and, crucially, belonging to the same country. In particular, our evidence suggests that political geography has been highly important for the diffusion of patenting till around two decades ago, slowly declining afterwards in favour of a more global technological innovation ecosystem. To this end, we improved current similarity-based prediction algorithms, i.e., algorithms based on the principle that the more two MAs are technologically similar, the higher the probability they will accomplish similar evolutionary technological paths. In particular, the improvement is substantial to forecast the so-called MAs technical “debut”, i.e., the first-ever patent produced by an MA with a given technological code, where current models cannot formulate predictions.

We further visualise the evolution of countries and metropolitan areas in a two-dimensional space of competitiveness and diversification. To this end, we adopted the UMAP dimensionality reduction algorithm^41^ to visualise the different technological paths of countries and MAs. We discover the existence of two main technological innovation pathways, discriminating between different strategies towards progress. For instance, “Western” countries and BRICS (Brazil, Russia, India, China, South Africa) countries follow very different routes in this space, which we can define in terms of distinctive technological traits.

The paper is organised as follows. Section “Data” describes the data used in this work. In sSection “Methods”, we introduce the methodologies used in our work, explaining the details of the similarity measures and testing procedures adopted. In section “Results”, we present the results discussing the relevance of political geography, i.e., belonging to the same country, to obtain better predictive results, in particular, to predict the emergence of a brand-new technology in the portfolio of a given MA. We also display the technological innovation pathways of countries and MAs. Finally, in section “Discussion”, we summarise the main results and highlight the hints the present work can give to future works addressing the questions arising from this study.

## Data

### Technology Codes and Metropolitan Areas (MAs)

We adopt the PATSTAT database (www.epo.org/searching-for-patents/business/patstat) to provide information about patents and technology codes. The database contains approximately 100 million patents registered in about 100 Patent Offices. Each patent is associated with a code that uniquely identifies the patent and a certain number of associated technology codes. The WIPO (World International Patent Office) uses the IPC (International Patent Classification) standard^18^ to assign technology codes to each patent. IPC codes make a hierarchical classification based on six levels called digits, which give progressively more details about the technology used. The first digit represents the macro category. For instance, the code Cxxxxx corresponds to the macro category “Chemistry; Metallurgy” and Hxxxxx to the macro category “Electricity”. Considering the subsequent digits, we have, for instance, with C01xxx, the class “Inorganic Chemistry” and with C07xxx the class “Organic Chemistry”.

For the metropolitan areas (MAs), we adopted a database (see next section) to match the unique patent identifier and its technology code to the corresponding MA. To geolocalise the patents, we adopted the De Rassenfosse et al. database^42^ that contains entries on 18.9 million patents from 1980 to 2014. This is the first dataset about first filing applications from around the world, organised according to the location of applicants, i.e., companies or laboratories. This information helps study the geography of technological innovation and understand the spatial distribution of patented inventions. The geolocalisation is performed by linking the postal codes of applicant addresses to latitude and longitude and, as a result, to countries, regions, and MAs. The database contains information about the first application and assigns multiple technology codes to patents with more than one. The data is sourced from PATSTAT, WIPO, REGPAT, and the Japanese, Chinese, German, French, and British patent offices. Finally, each patent has unique identifiers, technology codes, and geographical coordinates (latitude and longitude). More information about De Rassenfosse et al. and PATSTAT database can be found in the Supplementary Information.

### Data Preparation

To clean the data, the first step consists of associating the technology codes of a patent with a specific MA by matching latitude and longitude information for each patent with the MAs borders obtained by the Global Human Settlement Layer^43^. This way, we can select the patents within each MA’s boundaries with their technology codes. Once this operation is completed, it is possible to build, year by year, the bipartite network that links MAs to technology codes. We represent the bipartite networks through bi-adjacency rectangular matrices ${\textbf {V}}^y$ whose elements $V_{a,t}^y$ are integers indicating how many times a technology code *t* appeared in different patents in a given MA *a* in year *y*.

Our network features 2865 MAs connected to 650 4-digit technology codes. We decided to work with four digits instead of more or less because with the 4-digit we can have a technological resolution such that these are neither too similar nor too far apart. With more digits, we would have trivial results: for example, the 4-digit code A01C (Planting; Sowing; Fertilising) contains codes A01C-15 (Fertiliser distributors) and A01C-21 (Methods of fertilising). With fewer digits, we would have the opposite problem. In addition, multiple digits would have inherent problems with the PATSTAT database due to changes in database versions. Over time, new codes are born, or others are removed. The 4-digit choice appears as the most stable.

Our networks are represented by a set of matrices ${\textbf {V}}^y$ for each year, *y*, from 1980 to 2010. Each year *y* matrix element $V_{at}^y$ counts how many times, in the year *y*, the technology *t* appears in the MA *a*. Finally, we binarise the matrices ${\textbf {V}}$ simply using 0 as a threshold to obtain 30 ${\textbf {M}}^y$ matrices:

$$\begin{aligned} M_{at}^y = {\left\{ \begin{array}{ll} 1\ \ \ \text {if}\ \ \ V_{at}^y \ne 0\\ 0\ \ \ \text {if}\ \ \ V_{at}^y = 0 \end{array}\right. } \end{aligned}$$

We decided to apply this binarisation procedure instead of the standard approaches like Revealed Comparative Advantage (RCA)^44^ because we are interested to know which MA is adopting a given technology for the first time.

## Methods

### Similarity measures

By the term *Similarity*, we mean a measure of closeness between nodes in the same layer. In previous studies^45–47^, the similarity in the layer of items was used to study how an element of the layer of users may evolve in the future. For example, in^37^, the similarity between technologies was used to predict the future technology production of firms. In^46,47^, the similarity between products was used to predict countries’ future product exportation competitiveness. We can apply the general similarity measure defined in literature^48^ to our MA-technology networks as:

1 $$\begin{aligned} B^y_{tt'}= \frac{1}{N_1}\sum _a \frac{M^y_{at}M^y_{at'}}{N_2}, \end{aligned}$$

in the case of technology similarity (between items), or

2 $$\begin{aligned} B^y_{aa'}= \frac{1}{N_1}\sum _t \frac{M^y_{at}M^y_{a't}}{N_2}, \end{aligned}$$

in the case of similarity of MAs (between users). Here, $N_1$ and $N_2$ are two parameters through which it is possible to define several types of similarity.

The simplest type is called *co-occurrence*^48^, and it is defined putting $N_1 = N_2 = 1$. Given two nodes of the same layer, this measure counts how many common neighbour nodes they have in the other layer. In our case, we measure how many MAs do the technology *t* and $t'$ in the same year or how many technologies are done by both MAs, *a* and $a'$, in the same year. However, different similarity measures can be found in the literature based on the value given to $N_1$ and $N_2$. We define by $d_a = \sum _t M_{a,t}$ the diversification of the MA *a*, i.e., the number of technologies done by *a*, and by $u_t = \sum _a M_{a,t}$ the ubiquity of technology *t*, i.e., the number of MAs active in that technology sector. Among the broadest similarity measures used are:

- Technology Space (TS). This similarity is based on the Product Space of^45^ and it has $N_1 = \max (u_t, u_{t'})$ and $N_2 = 1$ (or $N_1 = \max (d_a, d_{a'})$ and $N_2 = 1$ in the MA layer). Using this type of normalisation, one gives a lower connection weight to those technologies done by many MAs;
- Resource Allocation (RA)^49^. This similarity is obtained with $N_1 = 1$ and $N_2 = d_a$ ($N_1 = 1$ and $N_2 = u_t$ for MA layer). It is used to modulate the contributions of common neighbours with high degrees. If a MA has high diversification, RA will penalise the link between its technologies, given the triviality of their link. If the MA makes all the technologies, it is a given that each technology is linked with all the others.;
- Taxonomy (TAX)^50^. For this similarity $N_1 = \max (u_t, u_{t'})$ and $N_2 = d_a$ ($N_1 = \max (d_a, d_{a'})$ and $N_2 = u_t$ for the MA layer). The Technology Space gives a higher similarity score to technology with a low ubiquity (i.e., technology done by a few MAs) and, consequently, bias towards them. However, the idea is that these complex technologies are done by MAs (a few numbers) that do approximately all the others. Consequently, it is impossible to justify a city’s path from non-complex technologies to complex ones. Normalising also for the diversification, we avoid this problem as we penalise low ubiquity scores and complex technologies are weighted more.

Following Hidalgo et al.^45^, we define the quantities:

3 $$\begin{aligned} \omega ^{tec}_{at} = \frac{\sum _{t'} M_{at'}B_{tt'}}{\sum _{t'} B_{tt'}}\ \ \ \ \ \ \ \ \ \ \ \omega ^{MA}_{at} = \frac{\sum _{a'} M_{a't}B_{aa'}}{\sum _{a'} B_{aa'}}. \end{aligned}$$

$\omega ^{tec}_{at}$ measures how much the technologies done by the MA *a* are similar to the technology *t*. $\omega ^{tec}_{at}$ is thus high if MA *a* develops technologies close to the technology *t* $\omega ^{MA}_{at}$, instead, measures how much a given technology *t* is spread among MAs similar to the MA *a*. $\omega ^{MA}_{at}$ is thus high if technology *t* is spread among MAs surrounding MA *A*).

Given these definitions, we can use $\omega ^{tec}_{at}$ ($\omega ^{MA}_{at}$) as a prediction score: the higher is $\omega ^{tec}_{at}$ ($\omega ^{MA}_{at}$), the higher the probability that an MA *a* will start developing the technology *t*.

### Testing procedure

Given a matrix ${\textbf {M}}^y$, one of our purposes is to predict the same matrix $\delta$ years later, ${\textbf {M}}^{y+\delta }$. The basic idea is that higher values in $\omega ^{tec}_{at}$ or $\omega ^{MA}_{at}$ will correspond to new technologies, i.e., more 1s, in ${\textbf {M}}^{y+\delta }$. To this end, we have to keep into account two elements.

- Class Imbalance. We are treating our problem as a classification one, i.e., we want to predict if an MA will make or not a given technology. Class labels, in our case, are 0s and 1s, but the number of elements equal to 1 is approximately only 5%. To treat this unbalance correctly, we adopted the Area Under the Precision-Recall Curve^51^.
- Autocorrelation. With the term *autocorrelation*, we mean that if an MA does or does not do a given technology in a specific year, with a high probability it will continue his current behaviour in the future. To avoid this problem, the evaluation is performed only for activations events, i.e., events in which the technology is not done in the year *y* and it is done at year $y + \delta$. This strategy allows the healing of autocorrelation problems. Furthermore, it helps us study the diffusion of the technological process. We are more interested, in fact, in understanding where a new technology will be triggered rather than knowing which ones will not.

## Results

### Predictions

#### Geographic proximity and country diffusion

**Figure 1:**
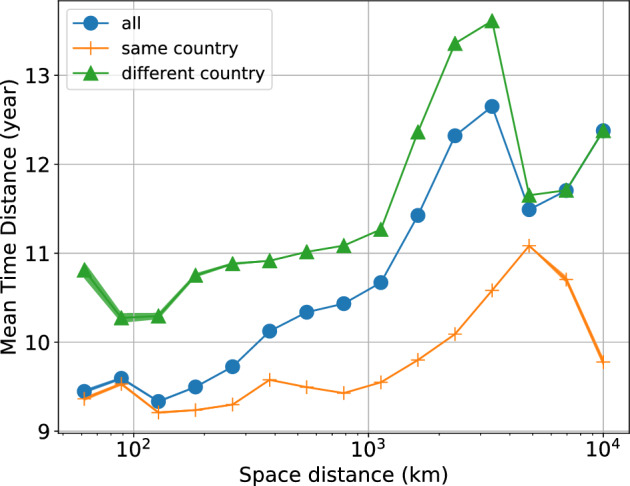
Mean time distance (see the text for the definition) aggregated on different spatial distance ranges belonging. The error for each beam is determined by calculating the mean standard deviation. Due to the significant number of points per beam, the error is often not visually discernible in most plots. The blue curve corresponds to the aggregation of all MAs. We observe the overall increase in the Mean Time Distance, signalling an important role of geographical distances. Second, we split the set of MAs into two subsets of pairs of MAs belonging (orange curve) or not (green curve) to the same country. The second important observation is that belonging to the same country greatly reduces diffusion times.

We analyse technology code diffusion timing to study the role of physical and political geography in technological innovation dynamics. Consider the MA where a specific technology code *t* first appears. We define the *Mean Time Distance* as the average time distance between the first appearance of *t* and its other first appearances in other MAs. After averaging over all technologies, we aggregate this mean on different spatial distance ranges to analyse the relationship with physical geography. On the other hand, to consider political geography, we calculate the average on the subsets of MAs belonging or not to the same country. In Fig. 1, we report our analysis on the Mean Time Distance.

Two important observations are in order. First, for the overall set of MAs, the Mean Time Distance increases on average with the geographical distance, signalling an important role of geography in the diffusion of technological innovation. Second, the Mean Time Distance is always shorter for the subset of MAs belonging to the same country, and it does not show a strong dependence from the spacial distance until the scale $10^3$ Km. After this scale, we see how a dependency from the spatial distance is stronger but more fluctuating (growing and then decreasing). This evidence is probably due to the distribution of MAs’ distances, which are affected by seas and oceans. In fact, until the scale $10^3$ Km, the distribution of distances (presented in Supplementary Information) follows a power law with exponent $\sim 2$, corresponding to an isotropic distribution in two dimensions. After that scale, the seas and oceans break the isotropy assumption, making the distribution less predictable and ultimately affecting Mean Time Distance. But also in this range, the MAs couples from the same country show a way lower Mean Time distance. Therefore, we can consider political geography as predominant over physical geography in the dynamics of technological innovation.

#### Role of countries: an improved model

In works concerning similarity and forecast on bipartite networks, it’s common to compute the prediction using the links between the items layer (technology codes, in our case), i.e., using $\omega ^{tec}_{at}$. However, mathematically, we have seen that it is possible to calculate a similarity between the nodes of both layers, i.e., also considering $\omega ^{MA}_{at}$. In the work of Albora et al.^52^, the authors show how a mean between the two scores can outperform the standard method. They also propose a linear combination of item-based and user-based estimations, showing how this method outperforms the others. In our case, to get the prediction, we utilised this last method, computing a linear combination of technology and MA densities instead:

4 $$\begin{aligned} S^{y+\delta }_{at} = \alpha \omega ^{tec}_{at} + \beta \omega ^{MA}_{at}. \end{aligned}$$

where $S^{y+\delta }_{at}$ is the forecast for the year $y + \delta$. If we consider MAs with no patent in the year *y*, regardless of the similarities used, the predictions obtained from $\omega ^{tec}_{at}$ and $\omega ^{MA}_{at}$ will always be zero by construction. This outcome is due to the presence, in the rows of ${\textbf {M}}$ matrices related to those MAs, of only 0s. Given the relevance of belonging to a country unveiled through our previous results, we included that information to predict when a given MA will start patenting a specific technology for the first time. To this end, we define:

5 $$\begin{aligned} \omega ^{C}_{at} = \sum _{a'}{M_{a't}^{y}\frac{C_{aa'}}{\sum _{a}{C_{aa}}}}, \end{aligned}$$

where $C_{aa'} = 1$ if *a* and $a'$ belong to the same country, 0 otherwise and $\sum _a C_{aa}$ is the number of MAs in the same country as *a*, inserted to avoid size effects. $\omega ^{C}_{at}$ represents the average values of technologies done by the MAs of a specific country. As explained in the Method section, the higher the value of $\omega ^{C}_{at}$ is, the higher the probability that $M_{at}^{y+\delta } = 1$.

Our prediction model is thus a linear combination of the three previous contributions: technology similarity, MA similarity and information on belonging to the same country:

6 $$\begin{aligned} S^{y+\delta }_{at} = \alpha \omega ^{tec}_{at} + \beta \omega ^{MA}_{at} + (1-\alpha -\beta )\omega ^{C}_{at}. \end{aligned}$$

Also in this case, the higher the value of $S^{y+\delta }_{at}$, the higher the probability to have $M_{at}^{y+\delta } = 1$. Because of the Autocorrelation problem explained in the Method section, we decided to evaluate our predictions on the so-called *activation* elements, i.e., the matrix elements $M_{at}^{y} = 0$ and that in $y+\delta$ could become 1.

**Figure 2:**
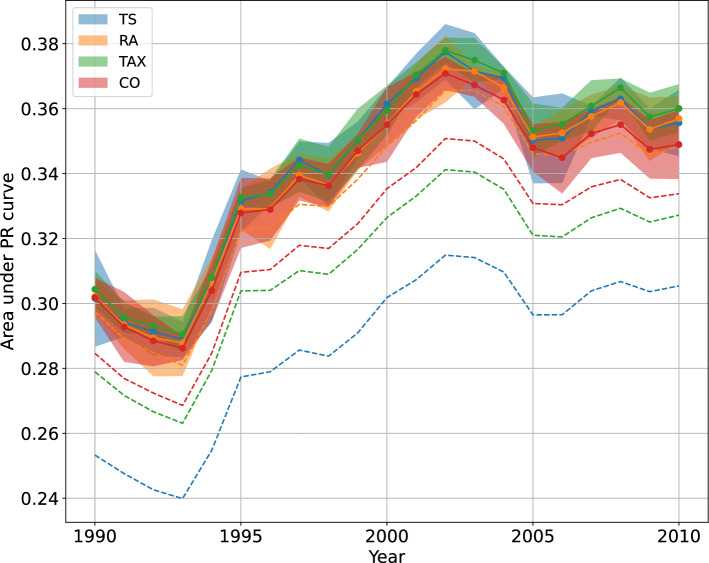
Performances of predictions models. Continue curves represent the prediction scores of our improved model (Eq. 6) for the four similarity metrics defined in the text: TS, RA, TAX and CO. For comparison, dotted curves report the same prediction scores of the classical model based on the item–item similarity $\omega ^{tec}_{at}$. Our improved model outperforms the classic approaches. Error ranges are obtained using a 5-fold cross-validation to select the best parameter values $\bar{\alpha }$ and $\bar{\beta }$ out-of-samples.

In Fig. 2, we compare the prediction for $\delta = 10$ of the four metrics of similarity defined above. We also compare our model (continue curves) and classic models, i.e., models using the items-items similarity $\omega ^{tec}_{at}$ (dotted lines). We can see how our model curves outperform all the dotted ones. In Supplementary Information, we also report the analysis done by using $\delta = 1$ and $\delta = 5$.

If we consider MA with no technologies in *y*, both $\omega ^{tec}_{at}$ and $\omega ^{MA}_{at}$ are 0 by definition. In this case, the predictions of our models are only due to $\omega ^{C}_{at}$, which represents the influence of countries.

In this specific case, we compared our results (Model) against a null model (Rand) and a model based on the spatial distance (Dist) to validate our findings. The null model prediction for each MA is a redistribution of the predicted technologies in the whole vector of the technological codes. If, for a given MA, we predict (0, 0, 1, 0), the null model would predict (0.25, 0.25, 0.25, 0.25). On the other hand, the spatial distance model uses geodetic distances between MA as similarities. In Table 1, we compare, for different values of $\delta$, the models’ performances on technological debuts of MAs by summing the areas under the curves for all years. Our model, informed on country membership, is the most successful in estimating future technologies made by an MA with a null technology portfolio.

**Table 1:** Models comparison.

	$\delta _1$	$\delta _5$	$\delta _{10}$
Model	0.075	0.094	0.138
Dist	0.052	0.079	0.102
Rand	0.017	0.032	0.076

In the table, we compare, for different values of $\delta$, the values of the areas under the curves of the predictions made on the MAs with zero technologies using the information belonging to the same country, geographic distances, and the random case. The belonging to the same country is the information that most successfully provides an estimate of future technologies made by an MA with 0 technology portfolio.

### Model analysis

**Figure 3:**
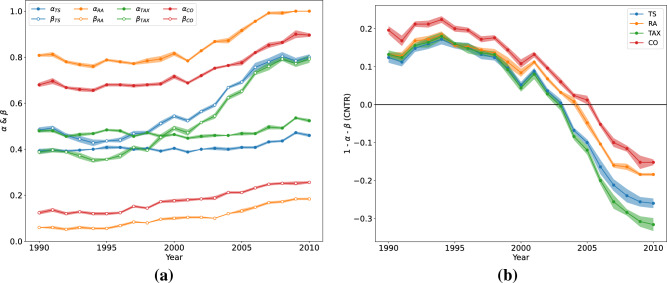
Analysis of model optimal parameters with $\delta =10$. (**a**) Optimal $\alpha$ and $\beta$ over the years for different similarity metrics. We can see how both started to increase around 2000. (**b**) The contribution of country information over the years, estimated as $1-\alpha - \beta$. We show how the contribution of country information is positive in the early years, but around the late 90s’, this tends to decrease and even become negative.

In this section, we analyse the behaviour of the best parameters $\alpha$ and $\beta$ over the years. For each metric, we show in Fig. 3a the optimal values of $\alpha$ and $\beta$ over the years considering $\delta =10$. In Supplementary Information, we have reported the same analysis for $\delta =1$ and $\delta =5$. In this figure, we can see a common trend. Both $\alpha$ and $\beta$ tend to stay constant till the end of the 90s’. After that, their values tend to increase, as all four similarity metrics predicted. This analysis is confirmed by the descending behaviour, in Fig. 3b, of the term $1-\alpha - \beta$, representing the importance of belonging to a country. These pieces of evidence suggest that political geography has been highly important for the diffusion of innovation till around two decades ago. After that, the evidence indicates that the overall ecosystem of MAs became more global and based more on similarities between technologies and MAs. At the beginning of our period of observation in our data, the country term $1-\alpha - \beta$ has a positive contribution, but around the end of the 90s’, it tends to decrease and even becomes negative. We interpret this result as a change in the dynamics of technological innovation in countries where the similarity between technologies and MA starts to become more important than belonging to the country itself. This is likely because, instead of following national trends, many MAs could have begun to copy MAs in other countries. This phenomenon can be explained by the loosening of institutional barriers to international mobility, with a resulting globalisation of labour markets. Thus, when we observe that the role that borders play is diminishing in the development and diffusion of new technologies, this is mainly due to the erosion of institutional frictions that hinder international connectivity^53^ and the strengthening of global collaboration networks^54^. Together with these mechanisms also the general market globalisation plays a role. In fact, the enhancement of competitiveness to a global scale probably creates collective dynamics, even when there is no cooperation but competition, instead. This will probably give rise to innovation trends diffusing at the global scale. These considerations imply that the development of new technology takes place simultaneously at the global level to win primacy in its production.

### The paths to technological innovation

In this last section, we focus on technological innovation paths, i.e., the paths followed by countries and metropolitan areas towards technological innovation. Though diversification is a good proxy for progress to technological innovation, we need another metric to represent similarities between the countries’ development strategies. We define, in particular, a metric that quantifies how competitive a country *c* is in a specific technology code *t* in year *y* relative to other countries, based on the number of MAs in *c* that patent with that technology code. Similarly, we can quantify how competitive an MA *a* is compared to other MAs. For each country, we define the following:

7 $$\begin{aligned} G_{ct}^y= \frac{C_{ct}/C_c}{C_{wt}/C_w}, \end{aligned}$$

**Figure 4:**
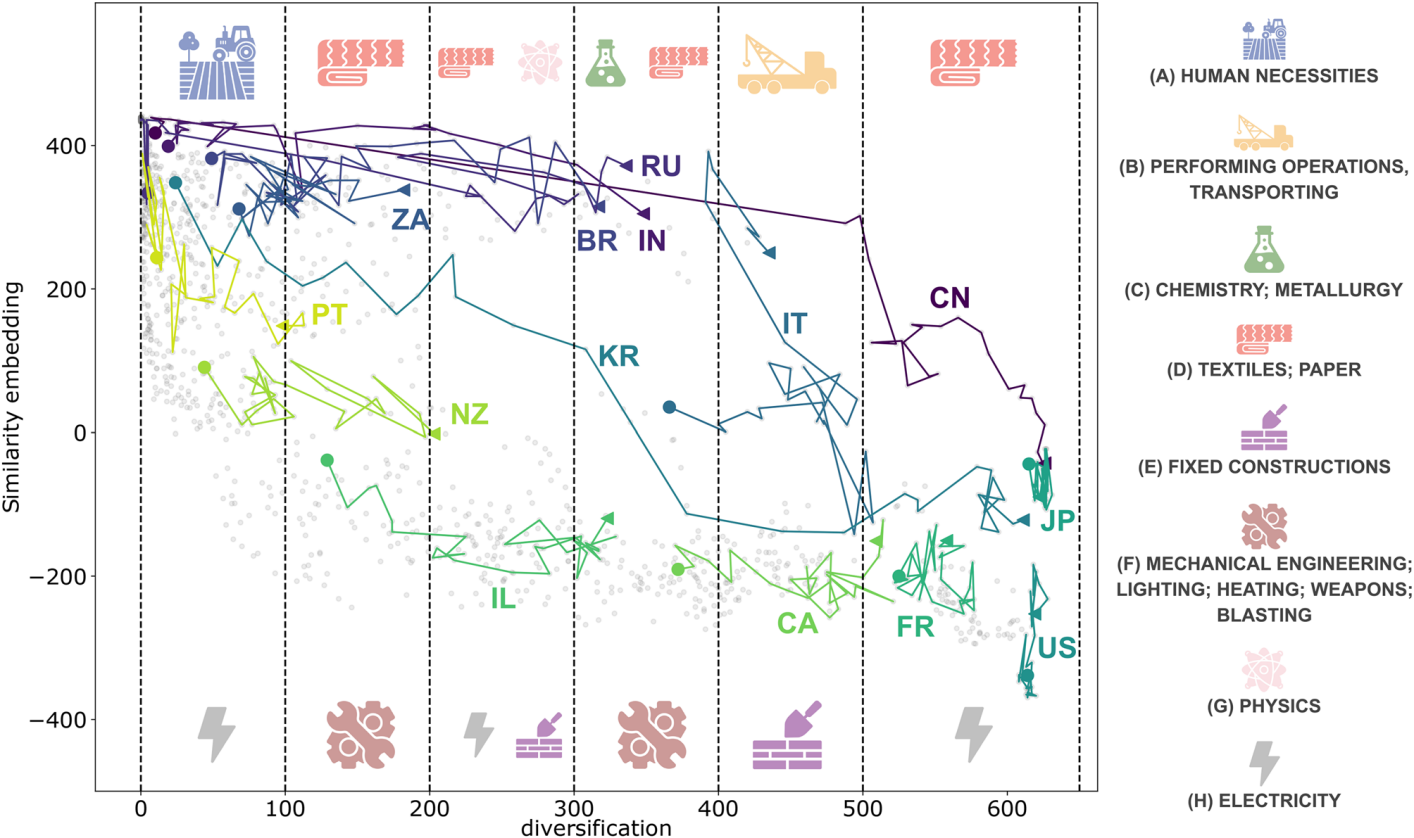
Country’s 1D similarity embedding versus diversification. Each point represents a country in a given year. For some countries, we plotted the trajectory over time. We can see how countries tend differently to reach a point of accumulation where the most developed countries are. In the lower part, we find the typical path of Western countries, and we report, for example, France, Canada, New Zealand and Israel. To highlight the technology difference between the “upper” and the “lower” paths, we divided the diversification into ranges of size 100 (except the last one). We focus on each range’s highest and lowest 25th percentile, aggregate the technologies to the 1st digit, and identify the most distinctive of the two subsets. The relative icons are reported on the top and bottom of each diversification range. The “upper” part is dominated mainly by the BRICS. In technological code terms, we can highlight the differences between the two extreme paths: the “upper” part dominates mostly in manufacturing technology as Textiles and Paper. The leftmost part, i.e., the least diverse, particularly dominates in technologies devoted to Human necessities. The “lower” part dominates in most sophisticated technologies such as Electricity, Fixed construction and Mechanical engineering.

**Figure 5:**
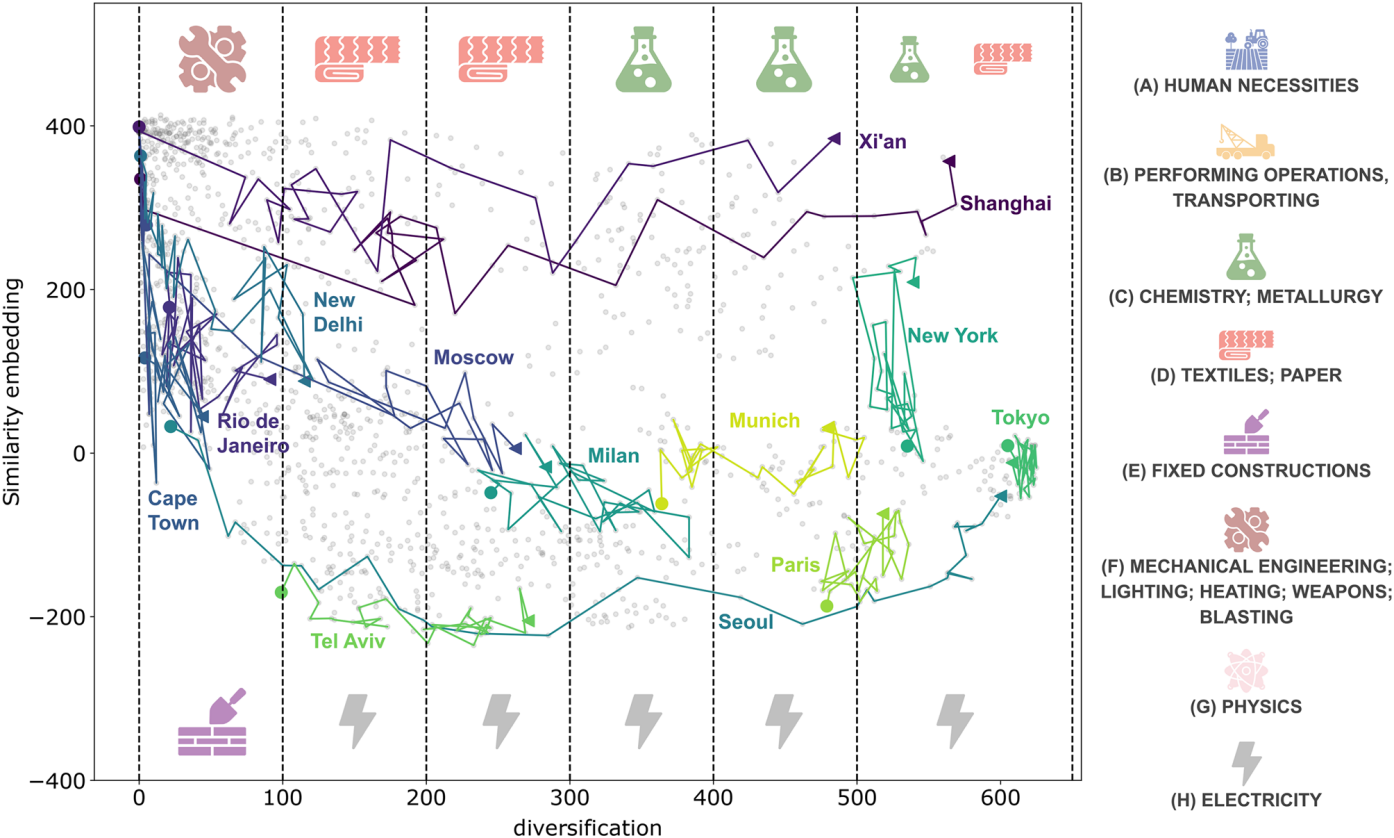
MA’s 1D Similarity embedding versus diversification. Each point represents an MA in a given year. To highlight the technology difference between each diversification range’s “upper” and the “lower” paths, we follow the same procedure of Fig. 4. The technology differences show that the lower path dominates in Electricity technologies, while the upper path dominates in Chemistry, Textiles and Paper technologies. We see how some MAs tend to chase others (Seoul vs. Tokyo, or Moscow vs. Milan), though, unlike the countries’ case, no single accumulation point emerges.

$C_{ct}$ counts how many MAs in the country *c* do the technology *t*, and $C_c$ is the number of MAs in the country *c*. $C_{wt}$ counts how many MAs are in the entire database patent with the technology code *t*, and $C_w$ is the total number of MAs. Therefore, $G_{ct}^y$ measures the fraction of MAs in *c* that do the technology *t* compared to the entire word for the year *y*. We define with $\bar{G}_{c}^{y}$ the vector that represents the average of $G_{ct}^y$ over all technologies *t*, and it represents the competitive position of the country *c* for the year *y*. Similarly, for each MA, we define the following:

8 $$\begin{aligned} G_{at}^y= \frac{M_{a\in c,t}}{C_{ct}/C_c}. \end{aligned}$$

and, similarly, $\bar{G}_{a}^{y}$ is the average of $G_{at}^y$ over all technologies *t* and it represents the competitive position of MA *a* for the year *y*. For every year, $G_{ct}^y$ and $G_{at}^y$ are vectors with 650 entries, corresponding to the total number of technologies. Using UMAP, we reduced the dimensionality to one and defined the similarity embedding. We found that this embedding is strongly anti-correlated with the modules of $G_{at}$ and $G_{ct}$ (see the Supplementary Information for further information). This evidence implies that the lower the similarity embedding, the higher the competitiveness of countries or MAs. We can thus use the similarity embedding as a reverse measure of competitiveness and plot the time evolution of each country and each MA in a two-dimensional scatter plot determined by the two quantities: similarity embedding (a reverse proxy for competitiveness) and diversification. We report the results in Fig. 4 for countries and Fig. 5 for metropolitan areas. Each point on the two plots is a pair country/year and MA/year.

We have highlighted the paths over time, followed by a selection of countries and MAs. Two typical patterns emerge that we denote as the “upper” path and the “lower” path. This pattern is particularly evident for countries. A country or MA that moves from left to right increases its diversification but not the competitiveness in the technologies that it does. Instead, movements from the upper part to the bottom are associated with growth in terms of competitiveness, keeping fixed diversification. The main difference between the two typical paths is the order of these movements. In the “upper” path, we first observe an increasing diversification and then an increase in competitiveness. In the “lower” path, the opposite occurs: first, an increase in competitiveness followed by a diversification increase. We coloured with different shades of the same colour the evolution of some countries belonging to the two typical paths.

Finally, to highlight the technology difference between the “upper” and the “lower” paths of both figures, we divided the diversification into ranges of size 100 (except the last one). For each range, we focus on the highest and lowest 25th percentile and aggregate the technologies to the 1st digit, representing the general technological category. We compare the technological categories present in the two sets to highlight the most distinctive ones, i.e., those with the greatest difference in rank based on their frequency in the subset. For instance, if a technological category *X* is the most common in the top 25% set and the least common in the bottom 25% set, *X* will be considered as distinctive of the top set while, if it had been the most common in both sets, it would not have been considered distinctive. See Supplementary Information for more details.

In Fig. 5, we show the results for MAs. Unlike countries, we do not observe a point of accumulation between MAs. We observe how some MAs get closer to others, such as Moscow to Milan, Seoul to Tokyo or Shanghai to New York. From a technological point of view, results are consistent with countries. The upper part is dominated by manufacturing technologies, while at the bottom one, there is more evident dominance of Electricity technologies.

Let us now focus on interpreting the different pathways in terms of strategies and policies implemented by countries and metropolitan areas. A case of particular interest is that of China, where R &D investment led to a patent boom^55–57^ and a consequent sudden increase in technological diversification. This sudden increase, however, was also paralleled by a deterioration in patent quality, as Dang et al.^55^ pointed out. This evidence explains why the path of China first presents an increase in diversification (i.e., moving rightward on the horizontal axis), followed by a later increase in competitiveness (i.e., moving downward along the vertical axis). While adopting distinct policies and behaviours as discussed in Lacasa et al.^58^, the other BRICS countries exhibit a similar trajectory to China. Lacasa et al.^58^ elucidated that high-income countries such as the EU15, the United States, and Japan are more actively involved in cutting-edge technologies than all BRICS economies. Still, China stands out of the BRICS countries since it managed to acquire a remarkable global influence in innovation, positioning itself as an innovation leader among the BRICS countries^59^, as demonstrated by Wang^60^.

In the most recent period, China was the only BRICS country to bridge the gap in frontier technology activities, reaching levels observed in high-income countries. The remaining BRICS economies have yet to narrow this disparity in frontier activities compared to high-income economies. Across all technological fields, BRICS economies exhibit a similar, low degree of diversification. Among them, Brazil stands out with the lowest degree of global interaction, while India appears relatively more engaged in generating patentable knowledge than other BRICS economies. Overall, the technological advancement profiles of the BRICS countries between 1989 and 1997 show an unexpected uniformity, reflecting their limited involvement in cutting-edge technology activities and a low level of integration with the global economy at that time. China stands out also because it significantly expanded its scale of activities in behind-the-frontier and frontier technology, boosting a high percentage of patents in high-tech domains and holding a solid position in technological knowledge diversification within the BRICS group.

Shifting now our focus to the “lower” part of chat diversification-competitiveness, Israel provides an illustrative example. Israel’s consistently high investments in R &D have propelled it into a technologically advanced nation, as highlighted by Beyar^61^. According to OECD data (available at https://data.oecd.org/rd/gross-domestic-spending-on-r-d.htm), Israel has steadily increased its gross domestic spending on R &D since the early 1990s, ultimately achieving the top ranking in investment by the year 2000. Its observed trajectory can be attributed to the substantial investments in R &D, which improves the quantity (diversification) and the quality (competitiveness) of technologies.

## Discussion

This study provides insights into technology diffusion among MAs worldwide and how geography impacts this process. Comparing geographic proximity, we find that belonging to a country is relevant in determining the likelihood of technology diffusion between metropolitan areas. Results indicate that, at equal geographical distances, technology diffusion occurs more readily across metropolitan areas belonging to the same country.

We develop a predictive model for future technology production of MAs that considers similarities between technologies and metropolitan areas and adds the contribution related to belonging to the same country. This last term allows for predictions even for metropolitan areas with empty technology portfolios. Our model outperforms traditional algorithms, particularly when one focuses on the case of technological debuts, i.e., when a metropolitan area starts developing a technology for the first time.

The study of the forecasts and the models’ parameters highlights the increasing importance of similarities between technologies and metropolitan areas as years pass. In particular, around the end of the 90s, belonging to a country lost its significance as a predictor of technological innovation paths in favour of the similarity among technologies and metropolitan areas. This finding suggests a change in the dynamics of technological innovation. To get a deeper insight into this phenomenology, we represented the temporal paths of MAs and countries in the technological space of innovations. This space comprises two dimensions, corresponding to technological competitiveness and the diversification of countries and metropolitan areas. We singled out two main paths, one followed by most Western countries and the other by the BRICS ones.

In Fig. 4, the presence of a main growth path (with countries such as New Zealand, Israel, France, etc.) is evident. In contrast, the upper part is dominated mainly by the BRICS economies. We can highlight the differences between the two paths in technology code terms: the upper part dominates mostly in manufacturing technology, such as Textiles and Paper. The leftmost part, i.e., the least diverse, particularly dominates Human necessities technologies. The lower part dominates in most sophisticated technologies such as Electricity, Fixed construction and Mechanical engineering. The two different paths differ not only in technological production but also in the significance of similarity embedding. As explained in the section ”The paths to technological innovation”, similarity embedding is related to the modulus of *G*. This sheds light on the two approaches, evidenced by the paths and fine-tuned by the belonging to different countries. In particular, the “upper” path first grows in diversification, and only then is there a change in embedding similarity. This implies that countries in this pathway aim first to develop new technologies and only then become world leaders. In contrast, the “lower” path does the opposite: countries belonging to this path develop few technologies in which they become leaders. Later, they get to develop new ones. It is also important to note that these pathways result from the patenting activity of the most developed cities in the selected countries (i.e., those that can patent). Consequently, these pathways do not consider less developed cities where other types of innovation are predominant, such as R &D investments^24,32^.

The model developed in this study can predict technology diffusion transparently and understandably, differently from other “black box” predictive models present in literature. These features allow for informed decision-making regarding investment and technological innovation. From this perspective, our scheme could be a valuable tool for policymakers to guide investment decisions and prioritise innovation areas.

On a scientific level, this study opens the door to future work and questions. First, starting from the model presented in this work, which is focused on activations, i.e., first occurrences of a given technology, one could generalise to predict also predict “shutdowns”, i.e., when a technological category is not patented any more. Furthermore, model simulation can be used to build green and sustainable pathways and highlight them at the level of MAs, regions, countries or companies. Another aspect that can be analysed is the study of innovation paths not only focused on technological innovation, then also considering other forms of innovation as defined in the work of Filippopoulos et al.^24^ and Rutten^23^. Finally, the relationship between forecasts and macroeconomic variables such as GDP can be explored to improve our understanding of technological innovation and economic dynamics.

Before concluding, it is essential to understand the limitations of the model. As stated in the introduction, the only use of patents as a proxy for innovation^20^ represents one crucial constraint. Inventions do not represent all forms of knowledge production in the economy, nor do patents cover all generated knowledge^21^. Other forms of innovation in cities and regions include diversity, cosmopolitan environment, creativity, inclusion, R &D and collaboration networks^23,24^. In particular, lagging regions prioritise “softer” innovation aspects and rely on public R &D, tolerance/inclusion, or collaboration networks, which can offset geographical disadvantages while patenting is less relevant in these regions. These other forms of innovation in cities or areas relate to softer inputs and outputs, and the mechanisms that spread this type of innovation are not necessarily geographically bounded. They could also depend on the network of knowledge and individual mobility^24,62–64^. Also, remote working and dispersed research teams can mitigate the concentration of innovation in urban areas^65–68^, and future studies linked with this should take those phenomena into account.

### Supplementary Information

Supplementary Information.

## Data Availability

The data supporting this study’s findings are available upon reasonable request from the authors.

## References

[1] Colbaugh R, Glass K (2012). Early warning analysis for social diffusion events. Secur. Inform..

[2] Kim K, Jung J-Y, Park J (2012). Discovery of information diffusion process in social networks. IEICE Trans. Inf. Syst..

[3] Brockmann D, Helbing D (2013). The hidden geometry of complex, network-driven contagion phenomena. Science.

[4] Melo HP (2021). Heterogeneous impact of a lockdown on inter-municipality mobility. Phys. Rev. Res..

[5] Mazzoli M, Gallotti R, Privitera F, Colet P, Ramasco JJ (2023). Spatial immunization to abate disease spreading in transportation hubs. Nat. Commun..

[6] Weil AR (2018). Diffusion of innovation. Health Aff..

[7] Lengyel B, Bokányi E, Di Clemente R, Kertész J, González MC (2020). The role of geography in the complex diffusion of innovations. Sci. Rep..

[8] Geroski PA (2000). Models of technology diffusion. Res. Policy.

[9] Comin D, Hobijn B (2010). An exploration of technology diffusion. Am. Econ. Rev..

[10] Comin, D., Hobijn, B. & Rovito, E. Five Facts You Need to Know About Technology Diffusion. NBER Working Papers 11928 (National Bureau of Economic Research, Inc, 2006). https://ideas.repec.org/p/nbr/nberwo/11928.html.

[11] Frietsch, R. et al. *The Value and Indicator Function of Patents. Studien zum deutschen Innovationssystem 15-2010, Expertenkommission Forschung und Innovation (EFI)* (Commission of Experts for Research and Innovation, 2010). https://ideas.repec.org/p/zbw/efisdi/152010.html.

[12] Griliches, Z. Patent statistics as economic indicators: A survey. In *R &D and Productivity: The Econometric Evidence* 287–343 (University of Chicago Press, 1998).

[13] Leydesdorff L, Alkemade F, Heimeriks G, Hoekstra R (2015). Patents as instruments for exploring innovation dynamics: Geographic and technological perspectives on “photovoltaic cells”. Scientometrics.

[14] Youn H, Strumsky D, Bettencourt LM, Lobo J (2015). Invention as a combinatorial process: Evidence from us patents. J. R. Soc. Interface.

[15] Hall, B. H., Jaffe, A. B. & Trajtenberg, M. *The NBER Patent Citation Data File: Lessons, Insights and Methodological Tools*. NBER Working Papers 8498 (National Bureau of Economic Research, Inc, 2001). https://ideas.repec.org/p/nbr/nberwo/8498.html.

[16] Strumsky, D., Lobo, J. & Van der Leeuw, S. *Measuring the relative importance of reusing, recombining and creating technologies in the process of invention*. SFI Working Paper 2011-02-003:23 (2011).

[17] Strumsky D, Lobo J, Van der Leeuw S (2012). Using patent technology codes to study technological change. Econ. Innov. New Technol..

[18] Fall, C. J., Törcsvári, A., Benzineb, K. & Karetka, G. Automated categorization in the international patent classification. In *ACM Sigir Forum*, vol. 37, 10–25 (ACM, 2003).

[19] Jun, S. Ipc code analysis of patent documents using association rules and maps–patent analysis of database technology. In *Database Theory and Application, Bio-Science and Bio-Technology: International Conferences, DTA and BSBT 2011, Held as Part of the Future Generation Information Technology Conference, FGIT 2001 in Conjunction with GDC 2011, Jeju Island, Korea, December 8–10, 2011. Proceedings*, 21–30 (Springer, 2011).

[20] Hall B, Helmers C, Rogers M, Sena V (2014). The choice between formal and informal intellectual property: A review. J. Econ. Lit..

[21] Arts S, Appio FP, Van Looy B (2013). Inventions shaping technological trajectories: Do existing patent indicators provide a comprehensive picture?. Scientometrics.

[22] Hall BH, Jaffe A, Trajtenberg M (2005). Market value and patent citations. RAND J. Econ..

[23] Rutten R (2019). Openness values and regional innovation: A set-analysis. J. Econ. Geogr..

[24] Filippopoulos N, Fotopoulos G (2022). Innovation in economically developed and lagging European regions: A configurational analysis. Res. Policy.

[25] Florida R, Adler P, Mellander C (2017). The city as innovation machine. Reg. Stud..

[26] Boschma R, Balland P-A, Kogler DF (2015). Relatedness and technological change in cities: The rise and fall of technological knowledge in us metropolitan areas from 1981 to 2010. Ind. Corp. Chang..

[27] Jacobs, J. *The Economy of Cities. A Vintage Book, V-584* (Random House, 1969).

[28] Leydesdorff L, Persson O (2010). Mapping the geography of science: Distribution patterns and networks of relations among cities and institutes. J. Am. Soc. Inform. Sci. Technol..

[29] Bank, W. *World Development Report 2019: The Changing Nature of Work* (Washington, DC, 2018).

[30] Glaeser, E. *Triumph of the City: How Our Greatest Invention Makes Us Richer, Smarter, Greener, Healthier, and Happier* (Penguin Press, 2012).

[31] Newman, P. & Kenworthy, J. *The End of Automobile Dependence* (Island Press, 2015).

[32] Shearmur, R. Urban Bias in Innovation Studies. In *The Elgar Companion to Innovation and Knowledge Creation* 440–456 (2017).

[33] Asratian, A. S., Denley, T. M. & Häggkvist, R. Bipartite Graphs and Their Applications, vol. 131 (Cambridge University Press, 1998).

[34] Lopezaraiza-Mikel ME, Hayes RB, Whalley MR, Memmott J (2007). The impact of an alien plant on a native plant-pollinator network: An experimental approach. Ecol. Lett..

[35] Fedriani JM, Wiegand T (2014). Hierarchical mechanisms of spatially contagious seed dispersal in complex seed-disperser networks. Ecology.

[36] Koskinen J, Edling C (2012). Modelling the evolution of a bipartite network-peer referral in interlocking directorates. Soc. Netw..

[37] Straccamore M, Zaccaria A, Pietronero L (2022). Which will be your firm’s next technology? Comparison between machine learning and network-based algorithms. J. Phys. Complex..

[38] Tacchella A, Cristelli M, Caldarelli G, Gabrielli A, Pietronero L (2012). A new metrics for countries’ fitness and products’ complexity. Sci. Rep..

[39] Straccamore M, Bruno M, Monechi B, Loreto V (2023). Urban economic fitness and complexity from patent data. Sci. Rep..

[40] Pavlopoulos, G. A. et al. Bipartite graphs in systems biology and medicine: a survey of methods and applications. GigaScience **7**, giy014 (2018). 10.1093/gigascience/giy014 10.1093/gigascience/giy014 PMC6333914 29648623

[41] McInnes, L., Healy, J. & Melville, J. Umap: Uniform manifold approximation and projection for dimension reduction. arXiv preprint arXiv:1802.03426 (2018).

[42] De Rassenfosse G, Kozak J, Seliger F (2019). Geocoding of worldwide patent data. Sci. Data.

[43] Schiavina, M., Moreno-Monroy, A., Maffenini, L. & Veneri, P. *Ghs-fua r2019a—ghs functional urban areas, derived from ghs-ucdb r2019a* (2015). Tech. Rep. (European Commission, Joint Research Centre (JRC), 2019). http:data.europa.eu/89h/347f0337-f2da-4592-87b3-e25975ec2c95.

[44] Balassa B (1965). Trade liberalisation and “revealed” comparative advantage 1. Manch. Sch..

[45] Hidalgo CA, Klinger B, Barabási A-L, Hausmann R (2007). The product space conditions the development of nations. Science.

[46] Albora, G., Pietronero, L., Tacchella, A. & Zaccaria, A. Product progression: A machine learning approach to forecasting industrial upgrading. arXiv preprint arXiv:2105.15018 (2021). 10.1038/s41598-023-28179-x PMC9880377 36707529

[47] Tacchella, A., Zaccaria, A., Miccheli, M. & Pietronero, L. *Relatedness in the era of machine learning*. arXiv preprint arXiv:2103.06017 (2021).

[48] Teece DJ, Rumelt R, Dosi G, Winter S (1994). Understanding corporate coherence: Theory and evidence. J. Econ. Behav. Organ..

[49] Zhou T, Lü L, Zhang Y-C (2009). Predicting missing links via local information. Eur. Phys. J. B.

[50] Zaccaria A, Cristelli M, Tacchella A, Pietronero L (2014). How the taxonomy of products drives the economic development of countries. PLoS ONE.

[51] Saito T, Rehmsmeier M (2015). The precision-recall plot is more informative than the roc plot when evaluating binary classifiers on imbalanced datasets. PLoS ONE.

[52] Albora G, Mori LR, Zaccaria A (2023). Sapling similarity: A performing and interpretable memory-based tool for recommendation. Knowl. Based Syst..

[53] Hoekman J, Frenken K, Tijssen RJ (2010). Research collaboration at a distance: Changing spatial patterns of scientific collaboration within Europe. Res. Policy.

[54] Morescalchi A, Pammolli F, Penner O, Petersen AM, Riccaboni M (2015). The evolution of networks of innovators within and across borders: Evidence from patent data. Res. Policy.

[55] Dang J, Motohashi K (2015). Patent statistics: A good indicator for innovation in china? Patent subsidy program impacts on patent quality. China Econ. Rev..

[56] Hu AG, Jefferson GH (2009). A great wall of patents: What is behind China’s recent patent explosion?. J. Dev. Econ..

[57] Li X (2012). Behind the recent surge of Chinese patenting: An institutional view. Res. Policy.

[58] Lacasa ID, Jindra B, Radosevic S, Shubbak M (2019). Paths of technology upgrading in the brics economies. Res. Policy.

[59] Dovgal O, Goncharenko N, Honcharenko V, Shuba T, Babenko V (2019). Leadership of China in the innovative development of the brics countries. J. Adv. Res. Law Econ..

[60] Wang Y, Li-Ying J (2014). How do the bric countries play their roles in the global innovation arena? A study based on uspto patents during 1990–2009. Scientometrics.

[61] Beyar R, Zeevi B, Rechavi G (2017). Israel: A start-up life science nation. The Lancet.

[62] Bunnell TG, Coe NM (2001). Spaces and scales of innovation. Prog. Hum. Geogr..

[63] Breschi, S., Lissoni, F. et al. *Mobility and Social Networks: Localised Knowledge Spillovers Revisited* (Università commerciale Luigi Bocconi, 2003).

[64] Boschma R (2005). Proximity and innovation: A critical assessment. Reg. Stud..

[65] Clancy, M. S. *et al.**The case for remote work*. Tech. Rep., (Iowa State University, Department of Economics Ames, 2020).

[66] Delventhal, M. & Parkhomenko, A. *Spatial implications of telecommuting*. Available at SSRN 3746555 (2020).

[67] Gupta, A., Mittal, V. & Van Nieuwerburgh, S. *Work from home and the office real estate apocalypse*. Available at SSRN (2022).

[68] Shearmur R (2012). Are cities the font of innovation? A critical review of the literature on cities and innovation. Cities.

