# Supplementary material for: The geography of technological innovation dynamics
Source: Sci Rep. 2023 Nov 29;13:21043. doi: 10.1038/s41598-023-48342-8 (PMC10687091; doi:10.1038/s41598-023-48342-8)
Supplement: Supplementary file 1 — Supplementary Information. [file 41598_2023_48342_MOESM1_ESM.pdf]

Supplementary Information

The geography of innovation dynamics.

Matteo Straccamore, Vittorio Loreto and Pietro Gravino

Contents

1 Patent Data and Geolocation Methodology 3

2 Distances distribution 3

3 UMAP for visualization of innovation diffusion 3

4 Predictions with different delay values. 4

5 Technology differences more information. 6

1 Patent Data and Geolocation Methodology

In this study, we utilize the comprehensive patent database provided by De Rassenfosse et al. [1], which offers geolocation information for approximately 18.9 million patent applicants. Our objective is to construct a dataset consisting of early patent applications from across the globe, organized according to the geographical locations of the applicants. This dataset enables us to identify the origins of patented inventions and locate the centers of innovation. The geographic information provided by this dataset proves valuable for investigating the geography of innovation, understanding the spatial distribution of patented inventions, and informing policymakers interested in firm location decisions and the attraction of highly skilled workers.
The authors of the database perform geolocation by associating postal codes of applicant addresses with latitude and longitude coordinates, thereby linking the applicants to their respective countries, regions, and cities/metropolitan areas. To obtain the postal code information, the authors match the addresses from the patent applications in PATSTAT and other databases, which, in turn, provide the corresponding postal codes.
Here are some pertinent features of this database:

- The patents included in this dataset represent the first application for a given invention.
- Some patents may have multiple geolocations due to collaborations among multiple applicants.
- Each patent is associated with one or more IPC 4-digit technology codes. In cases where a patent is associated with multiple technology codes, we retain all of them. This approach helps preserve the granularity of the data, as the technology categories are already quite specific.
- As the database focuses on the first application of a patent, when a patent is shared across multiple patent offices, the applicant's position remains consistent.
- The geolocation information for all patents in the De Rassenfosse database is obtained from PATSTAT, WIPO, REGPAT, and the patent offices of Japan, China, Germany, France, and the United Kingdom.

2 Distances distribution

In order to gain a deeper understanding of the patterns depicted in Fig. 1 of the main text, we will now illustrate the distribution of distances in Fig 1. Observing the graph, we can discern that up to a distance of approximately 10^3 km, the distribution exhibits a relatively weak reliance on spatial distance. However, beyond this threshold, the influence of spatial distance becomes more pronounced yet remains somewhat erratic, fluctuating with both increases and decreases. Within the range, the distance distribution follows a power law with an exponent of ~ 2, indicative of an isotropic distribution within two dimensions. However, once this scale is surpassed, the presence of seas and oceans disrupts the assumption of isotropy, rendering the distribution less predictable. In the Figure, we plot also as a red line the fit of the distribution until 10^3 km. The resulting angular coefficient is 1.98.

3 UMAP for visualization of innovation diffusion

UMAP [2] is a dimensionality reduction technique that is used to map high-dimensional data to a lower-dimensional space while preserving the structure of the data as much as possible. It uses a combination of techniques from algebraic topology and Riemannian geometry to construct a low-dimensional representation of the data, which can be useful for visualization and exploration. UMAP has been shown to be competitive with other dimensionality reduction techniques such as t-SNE [3]

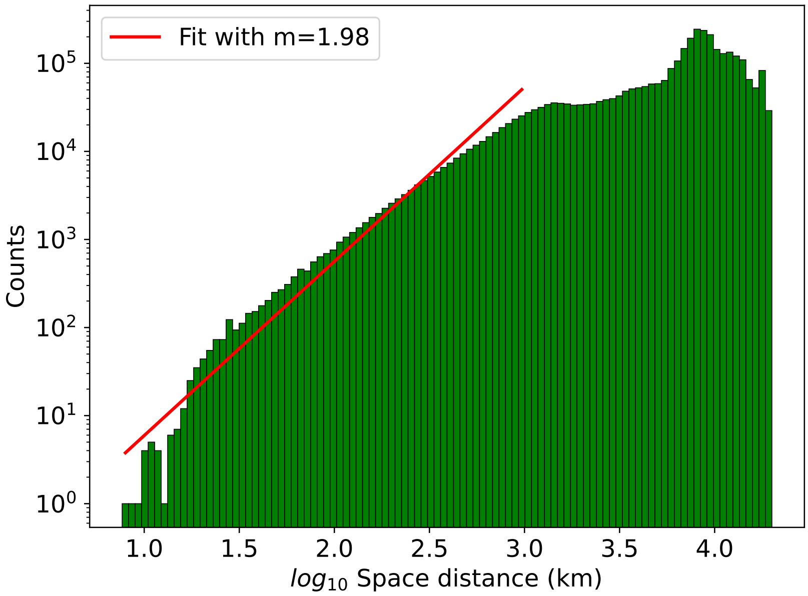

Figure 1: **Distances distribution**. Distances distribution in log-log scale. Up to a distance of approximately 10^3 km, the distribution exhibits a relatively weak reliance on spatial distance. However, beyond this threshold, the influence of spatial distance becomes more pronounced yet remains somewhat erratic, fluctuating with both increases and decreases. Within the range, the distance distribution follows a power law with an exponent of ~ 2, indicative of an isotropic distribution within two dimensions. The red line represents the fit of the distribution until 10^3 km. The resulting angular coefficient is 1.98.

and PCA [4], while preserving more of the global structure of the data.
Here we describe the parameter that we compute to find the best representation:

- **n_components**: This parameter controls the number of dimensions in the low-dimensional representation. We set this parameter equal to 1.
- **metric**: This parameter determines the distance metric used for the data. We chose to use the Cosine metric;
- **n_neighbors**: This parameter controls the number of nearest neighbours used to construct the low-dimensional representation. A larger value will preserve more of the local structure of the data, while a smaller value will result in a more global view of the data. The optimal value for this parameter will depend on the size and structure of the data. We put this value to 50;
- **min_dist**: This parameter controls the minimum distance between points in the low-dimensional representation. A larger value will result in more spread-out points, while a smaller value will result in more densely packed points. This parameter can be used to control the level of clustering in the low-dimensional representation. We put this value equal to 0;
- **spread**: This parameter controls the degree of freedom of the low-dimensional representation. A larger value will result in a more evenly distributed representation, while a smaller value will result in a more tightly packed representation. This parameter can be used to control the level of clustering in the low-dimensional representation. We put this value to 1000;

4 Predictions with different delay values.

In this Section, we present the prediction results setting the δ equal to 1 and 5. In general, the results are coherent with each other. In Fig.2 we show in each column respectively $\delta = 1$ and 5; each figure

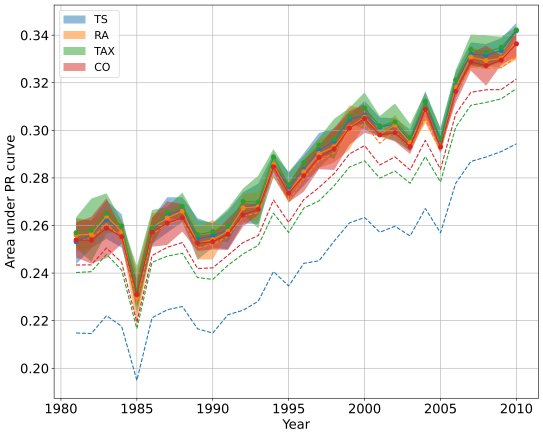

(a)

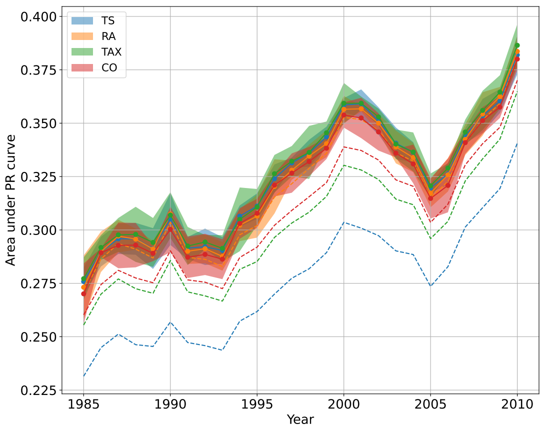

(b)

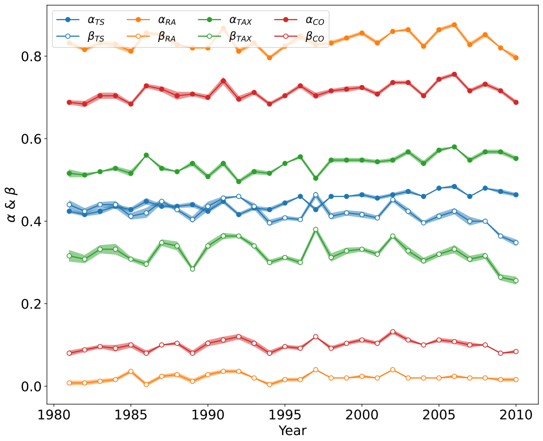

(c)

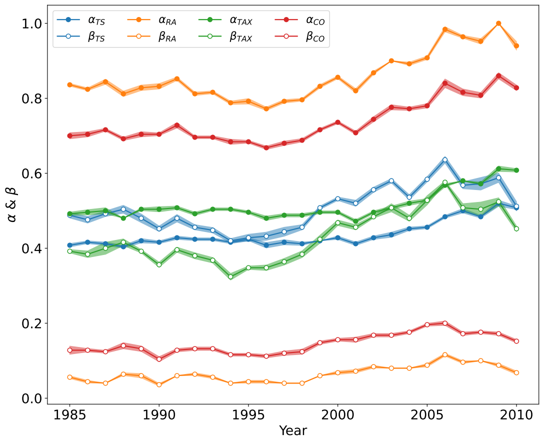

(d)

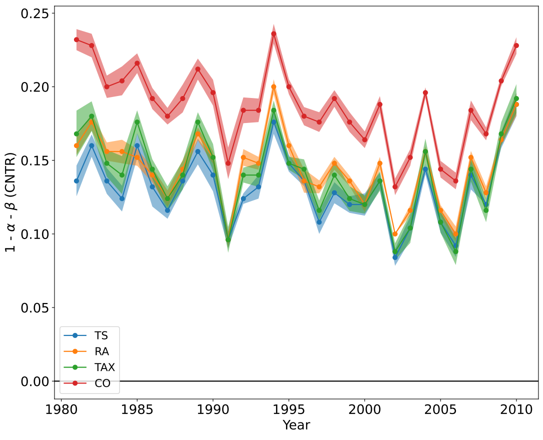

(e)

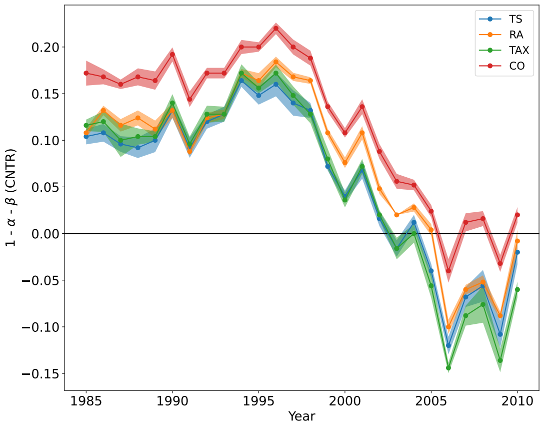

(f)

Figure 2: **$\delta = 1$ e $\delta = 5$ predictions with parameters analysis. (a-b)**: Predictions with $\delta = 1$ and 5 respectively. In the case of $\delta = 1$ we notice stability in parameter dependence, while already for $\delta = 5$ we get consistent results with $\delta = 10$ of the main text. The scores at $\delta = 1$ are still lower than at $\delta = 5$ and $\delta = 10$, and this is consistent with the result shown in Fig.1 of the main text in which we show that on average the time to the diffusion of technologies is about 10 years. **(c-d)**: Parameter trends of $\delta = 1$ and 5 respectively. While in the figure of $\delta = 5$, we notice similar but less pronounced behaviour than $\delta = 10$, $\delta = 1$ shows stable parameter values. **(e-f)**: Country importance of $\delta = 1$ and 5 respectively. Also in this case, for $\delta = 5$ we notice similar but less pronounced behaviour than $\delta = 10$. Instead for $\delta = 1$, we show an almost stable behaviour.

row is referred to predictions, parameter trends and country importance. The $\delta = 5$ case presents a similar but less pronounced behaviour than $\delta = 10$. Instead, $\delta = 1$ appears more stable, especially in terms of parameters. However, we can see that the scores at $\delta = 1$ are still lower than at $\delta = 5$ and $\delta = 10$, and this is consistent with the result shown in Fig.1 of the main text in which we show that on average the time to the diffusion of technologies is about 10 years.

5 Technology differences more information.

In this section, we show the intermediate steps used to obtain information about the technology diversification strategies of countries and MAs in the main text. For each range of diversification of Fig.4, we select the 25% of "upper" and "lower" points in both countries and MAs case. We show as an example the country case in Fig3. For each group, we calculate the z-score for each 3D-technology code to highlight the technology specialization of the two groups. In Fig.4, we plot the z-scores of "upper" and "lower" parts with respect to each technology relative to each diversification range of the previous figures. Here, we calculate the centroid for each macrocategory (i.e. 1D-technology). Finally, to highlight the higher differences between the two groups, we select the first 1D technology for each group such that the ranking difference is higher. As example, in Fig4**f**, the ranking for the 25% "upper" points is G:8, D:7, C:6, B:5, F:4, H:3, A:2, E:1; instead for the 25% "lower" points is H:1, F:2, A:3, G:4, B:5, E:6, C:7, D:8. The relative ranking distances between the eight macrocategories is, from A to H: -4, 1, 4, 6, -2, -3, 3, -5. This means that the "upper" part is more distinct with respect to the "lower" one in the D and the lower one in the H. It's important to note that the z-scores of the "lower" part are higher than the "upper" one; in fact, the "lower" countries are more technologically advanced. Here, however, we want to show the technological differences that the different parts focus on in relation to each other to better highlight the technological paths that distinguish the two parts.

References

1. Gaétan De Rassenfosse, Jan Kozak, and Florian Seliger. Geocoding of worldwide patent data. *Scientific data*, 6(1):1–15, 2019.

2. Leland McInnes, John Healy, and James Melville. Umap: Uniform manifold approximation and projection for dimension reduction. *arXiv preprint arXiv:1802.03426*, 2018.

3. Laurens Van der Maaten and Geoffrey Hinton. Visualizing data using t-sne. *Journal of machine learning research*, 9(11), 2008.

4. Andrzej Maćkiewicz and Waldemar Ratajczak. Principal components analysis (pca). *Computers & Geosciences*, 19(3):303–342, 1993.

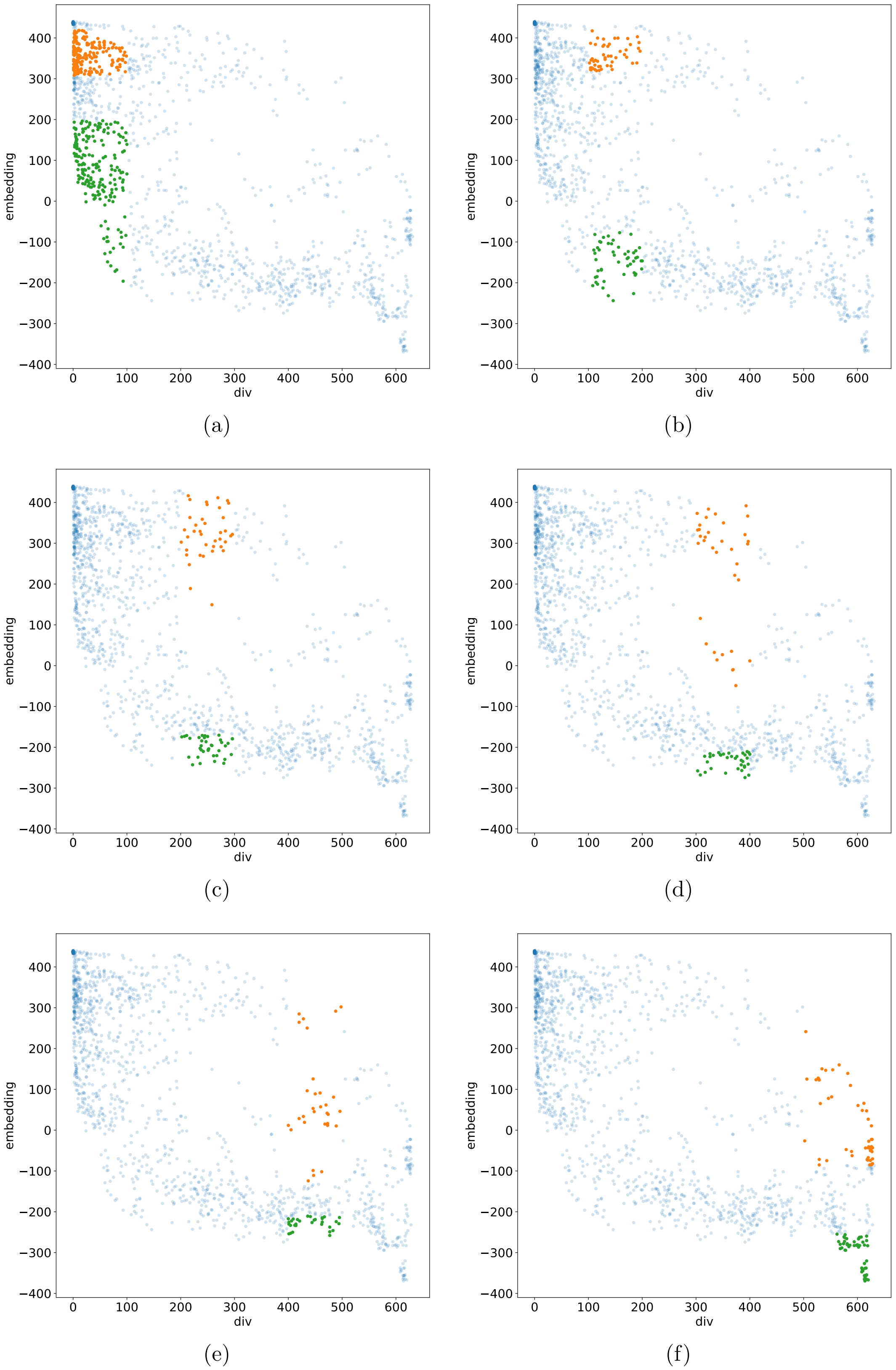

Figure 3: **Selection procedure of “upper” and “lower” points**. In each figure, orange points are the 25% starting from the upper to the lower, and the opposite is true for the greens. For the “upper” part, we decided to select the points just below the maximum disease because of the presence of country/year with 0 technologies.

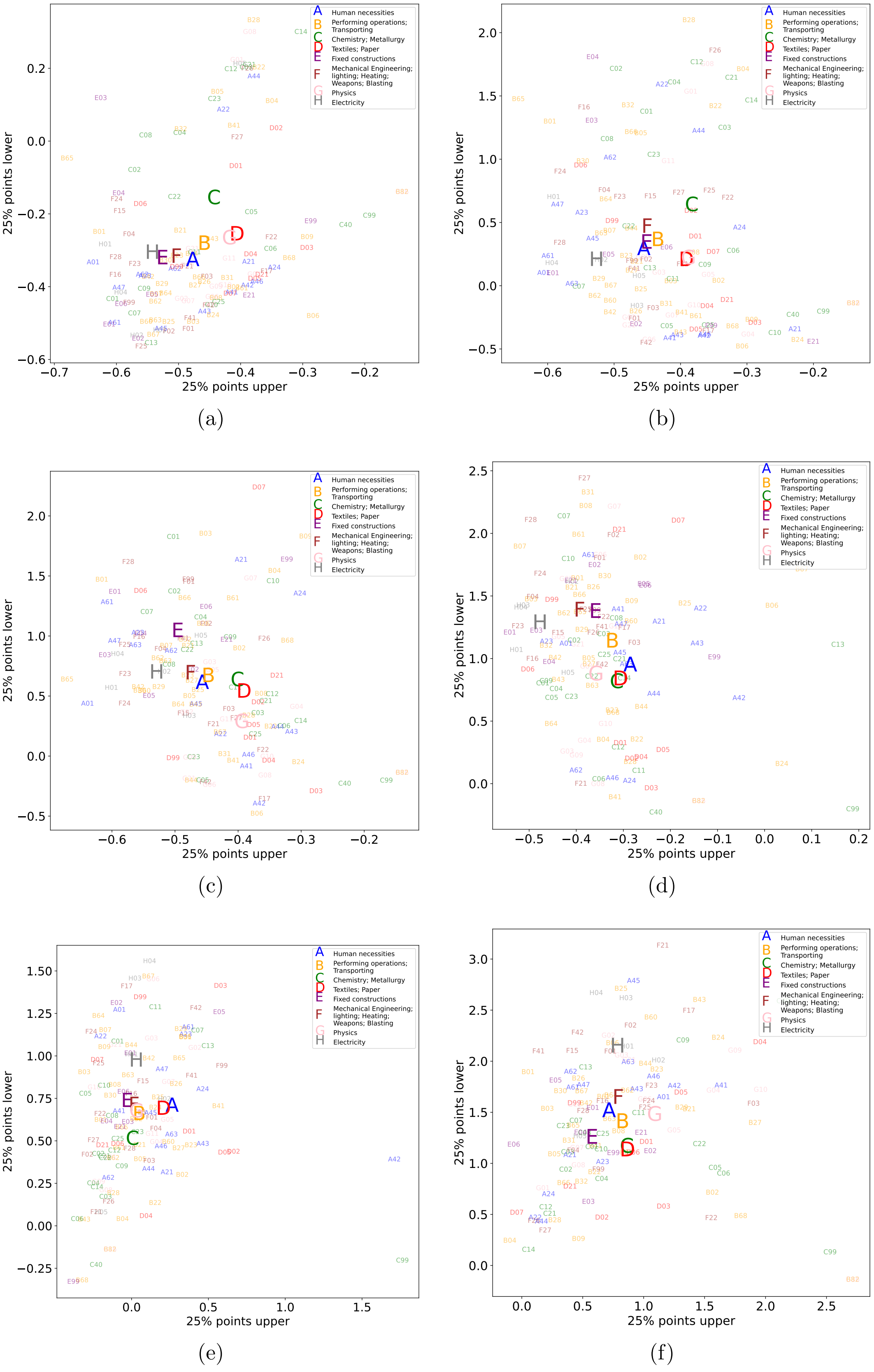

Figure 4: **Z-score of 3D-technology codes for "upper" and "lower" parts**. Each text is referred to as a 3D-technology code, and each colour is referred to as one of the eight macrocategory. We compute also the centroid of each 3D code plotting the respective 1D code.
